# Associations Between Psychological Profiles and Performance Success Among Professional Taekwondo Athletes in China: A Multidimensional Scaling Profile Analysis

**DOI:** 10.3389/fpsyg.2020.00822

**Published:** 2020-05-15

**Authors:** Bing Li, Cody Ding, Fenghui Fan, Huiying Shi, Liya Guo, Feng Yang

**Affiliations:** ^1^Center for Sport Psychology and Education, College of Sport Science, Southwest University, Chongqing, China; ^2^Education Sciences and Professional Programs, University of Missouri–St. Louis, St. Louis, MO, United States; ^3^Faculty of Psychology, Southwest University, Beibei, China

**Keywords:** psychological profiles, Taekwondo athletes, multidimensional scaling profile analysis, emotional traits, performance success

## Abstract

Sport psychology research has long sought to uncover the determinants of the optimal psychological state for peak performance. Persistent inquiries in this work include whether there is a set of ideal psychological and emotional factors that are required to achieve optimal performance and, if there are, what are they and how are they related to optimal performance. To answer these questions, the current study aimed to identify potential profiles of personality and emotional traits based on a sample of professional Taekwondo athletes from China. In addition, the study also aimed to examine the utility of the profiles in predicting successful athlete performance. Using multidimensional scaling (MDS) profile analysis, two latent profiles of personality and emotional traits were identified that indicate four subtypes of athletes. Regression analyses were conducted to examine how the identified profiles were associated with performance success. The results seemed to suggest that Taekwondo athletes with more performance success were more likely to have a profile of positive personality and emotional traits, while athletes with less performance success were likely to have somewhat elevated levels of self-control, extraversion, and aggression. Knowledge of athletes’ personality profiles will help sport psychologists develop suitable interventions to enhance athletes’ performance success. In concluding, the results are discussed in the context of athlete psychosocial development. The study added further evidence about the association between psychological and emotional factors and successful Taekwondo athletes.

## Introduction

All athletes strive to achieve peak performance, particularly during competition. For our purposes, it is important to note that “peak performance” is the state in which athletes can achieve superior functioning at their optimal psychological and physical levels, leading to outstanding outcomes (Chiodo et al., 2011; Harmison, 2011). The search for what leads to an optimal psychological state for peak performance has been a topic in sport psychology since 1977, following Ravizza (1977) work on peak performance. Questions such as whether intense training results in a particular athletic personality or psychological states (e.g., anxiety, depression, anger, hostility, fatigue-inertia, and low self-confidence) have long been investigated (e.g., Beall, 1986; Henschen, 1986; Morgan et al., 1987; Casolino et al., 2012). Further questions remain regarding what constitutes a psychological state conducive to consistently superior athletic performance during competitions (Gardner, 2009). As indicated by Anderson et al. (2014), research on the psychological factors of superior performance is critical for steering the courses of recruiting, training, and making interventions for professional athletes.

Some persistent questions in sport psychology include: whether there is there a set of ideal psychological and emotional factors for optimal performance? If there is, what are these ideal factors? Moreover, what are their links with optimal performance (Gould et al., 2002) More recent literature has explored diverse psychological variables (e.g., Krane et al., 2006; Casolino et al., 2012). For example, Ferrell et al. (2006) have reported that the psychological state comprised of relaxation, ease, calmness, automatic execution, and not being conscious of the moment is related to peak performance among athletes. We refer to this psychological state as “the zone of zero gravity” because it signifies a state in which athletes are not being pulled into anxiety, fear, or distraction.

Theoretically, if we assume that the zone of zero gravity is necessary for optimal performance and likely to be experienced by successful athletes, then it is worth investigating whether athletes with certain personality and emotional profiles are more likely to achieve this state. The earliest investigation into this question was done by Morgan and his colleague (Morgan, 1978, 1980). They found a mental health profile model in which more successful athletes exhibited few negative moods, such as tension, anger, depression, fatigue, and confusion. More recently, Casolino et al. (2012) found that athletes’ psychological state resembled the iceberg profile as assessed by the Profile of Mood State (POMS) (McNair et al., 1971) with a high level of one positive state (i.e., vigor-activity) and a low level of five negative states (i.e., depression-dejection, tension-anxiety, anger-hostility, fatigue-inertia, and confusion-bewilderment), although POMS did not differentiate between elite athletes in their study.

Meta-analyses have shown that personality traits (e.g., five personality dimensions) are associated with various personal, interpersonal, and social behaviors (e.g., Carver and Connor-Smith, 2010; Oh et al., 2011). One would also expect personality to predict sport performance (Allen et al., 2013). For example, Woodman et al. (2010) found that personality affects training effectiveness among British gymnasts, with conscientiousness predicting the quality of preparation, extraversion predicting distractibility, and emotional stability predicting the ability to cope with adversity. However, empirical findings in this regard vary. Studies based on large samples found that elite athletes are more extraverted and emotionally stable than recreational athletes (e.g., Egloff and Jan Gruhn, 1996) and, moreover, that athletes competing in national or international games have lower levels of neuroticism and higher levels of conscientiousness and agreeableness than those competing in club or regional games (Allen et al., 2011). However, the personality characteristics of novice athletes and experienced athletes do not meaningfully differ (e.g., Garland and Barry, 1990). Moreover, personality measures do not reliably predict single-match success (e.g., Rogulj et al., 2006). Studies comparing professional and non-professional athletes found that personality only has a small effect on season-long performance (e.g., Sindik, 2010), but that personality does have a large effect on progression to an elite professional level (Aidman, 2007; Gee et al., 2010; Martin et al., 2011).

Another interesting issue in sport psychology important to note for our purposes is athlete aggression and mental health. Reviewed literature suggested that antisocial behaviors in athletes, such as aggression, may influence emotion and performance during competition (Al-Yaaribi et al., 2017). For instance, anger has been reported to be negatively associated with concentration level (e.g., Silva, 1979). More recent studies suggest that depression and anxiety are salient issues among athletes (Drew and Matthews, 2017; Lebrun et al., 2018) a finding also evident in studies using POMS (Chiodo et al., 2011; Casolino et al., 2012). Based on these studies, one would expect mental health problems to be negatively associated with performance.

According to a review by Allen et al. (2013), these results are seemingly the most salient after more than 50 years of research. In recent years, the relationship between personality traits and athletic performance has re-emerged as an important topic; however, there currently remains more gaps in sport literature on this topic than meaningful findings (Allen et al., 2013). Collectively, the extant data on the topic seems to show that personality characteristics, along with emotional traits, such as depression, anger, impulse control, and aggression, can play an important role in sport performance. However, more research is needed to provide further information on how associations between these personality and emotional traits facilitate or hinder sport performance. Thus, the purposes of this study are (1) to identify personality and emotional trait profiles based on a sample of professional Taekwondo athletes and (2) to investigate the association between the identified profiles and performance success. Specifically, the first study aim was to provide a comprehensive picture of how personality traits (i.e., big five personality traits), along with emotional traits (e.g., aggression, depression, anger, envy, impulse control, and athlete self-control), act together among professional athletes by exploring profiles comprised of these variables using a sample of professional Taekwondo athletes from China. The second aim was to determine whether these profiles have any predictive power over performance success.

This study contributes to sport psychology literature in three ways. First, although personality and emotional traits are known to be associated with sport performance, our knowledge in this regard is still incomplete and gaps in literature remain. For example, Gardner (2009) contended that knowledge regarding psychological mechanisms underlying performance enhancement is lacking. Further research into psychological attributes that contribute to optimal performance is an important step in better understanding superior functioning during competition. By identifying professional athletes’ profiles of personality and emotional traits, we may obtain a basic understanding of the psychological characteristics of Taekwondo athletes. Second, a large proportion of studies on sport psychology have been conducted in the West. Few studies have been conducted on performance in an elite athlete population with experience in competition at international and national levels, particularly among Taekwondo athletes. The study will be the first to investigate the psychological characteristics of this population, and may provide information helpful for recruiting and training Taekwondo athletes to achieve an optimal psychological state at the highest level of professional competition. Third, the existence of an athletic personality remains an important concern in literature (Allen et al., 2013). Studying profiles of personality and emotional traits among professional athletes can provide valuable information on whether athletic profiles exist. In addition, in this study, we examined profiles of personality and emotional traits among athletes with different success levels within the same sport (i.e., Taekwondo). The findings may provide evidence regarding individual differences in the personality and emotional profiles of successful and less successful athletes. The study findings may prove important in informing future research and practice in sport psychology.

## Materials and Methods

### Participants

The data for the study included 332 professional Taekwondo athletes from different regions in China (187 males and 145 females). Participant age ranged from 16 to 30 years old, with mean age 19.32 (*SD* = 2.97) years. Experience in the sport ranged from 1 to 20 years, with the mean 5.81 (*SD* = 3.48) years. The weight category level ranged from 42 kg to 87 kg. Among the participants, 14 were elite athletes at an international level, 85 were elite athletes at a national level, 143 were first-level athletes at a national level, 55 were second-level athletes at a national level, and 35 were professional athletes without rank. All participants had not previously used psychological services.

### Measures

We used personality and emotional trait variables in profile analysis. We describe these measures in detail below.

#### Big Five – 44 (BIF – 44)

The big five personality traits were assessed using the Big Five Inventory – 44 (John and Srivastava, 1999). The measure has 44 items that are designed to assess extraversion (e.g., “I feel comfortable around people”), agreeableness (e.g., “I feel others’ emotions”), conscientiousness (e.g., “I pay attention to details”), openness (e.g., “I am quick to understand things”), and neuroticism (e.g., “I get stressed out easily”) on a 5-point rating scale ranging from 1 (*disagree strongly*) to 5 (*agree strongly*). A high score indicates a high level of the personality trait. Reliability, as assessed through Cronbach’s alpha, was 0.78 for extraversion, 0.79 for agreeableness, 0.81 for conscientiousness, 0.76 for openness, and 0.77 for neuroticism in the current sample.

#### The Aggression Questionnaire

Athlete aggression tendency was assessed through the Aggression Questionnaire (Buss and Perry, 1992). The measure comprises 29 items with four scales, namely: physical aggression (e.g., “Once in a while, I can’t control the urge to strike another person”), verbal aggression (e.g., “When people annoy me, I may tell them what I think of them”), anger (e.g., “Some of my friends think I’m a hothead”), and hostility (e.g., “At times I feel I have gotten a raw deal out of life”). Participants rated each item on a scale ranging from 1 (*extremely uncharacteristic of me*) to 5 (*extremely characteristic of me*). A high score indicates a higher tendency of aggression. Reliability, as assessed through Cronbach’s alpha, was 0.86 for physical aggression, 0.83 for verbal aggression, 0.77 for anger, and 0.87 for hostility in the current sample.

#### Depression

Depression was assessed through the Center for Epidemiologic Studies Depression Scale (CES-D) (Radloff, 1977). It is a 20-item instrument designed for screening depressed mood in the general population, covering the following areas: depressed mood, feelings of guilt and worthlessness, feelings of helplessness and hopelessness, psychomotor retardation, loss of appetite, and sleep disturbance. Participants rated each item on a scale of 1 (*rarely or none of the time*) to 4 (*most or all of the time*). Examples items are, “I had trouble keeping my mind on what I was doing” and “I talked less than usual.” Reliability, as assessed through Cronbach’s alpha, was 0.91 in the current sample.

#### UCLA Loneliness Scale

Perceived loneliness was assessed through the UCLA Loneliness Scale (version 3) (Russell, 1996). It is a 20-item scale designed to measure subjective feelings of loneliness and social isolation. Participants rated each item as O (*I often feel this way*), S (*I sometimes feel this way*), R (*I rarely feel this way*), or N (*I never feel this way*). Examples items are, “My interests and ideas are not shared by those around me” and “No one really knows me well.” Reliability, as assessed through Cronbach’s alpha, was 0.91 in the current sample.

#### Envy

A 7-item questionnaire was used to assess participants’ feelings of envy (Tandoc et al., 2015). Items are rated on a 5-point Likert scale, with 1 indicating *completely disagree* and 5 indicating *completely agree*. Example items are, “I generally feel inferior to others” and “It is so frustrating to see some people always having a good time.” High scores indicate a high level of envy. Reliability, as assessed through Cronbach’s alpha, was 0.78 in the current sample.

#### Athlete Self-Control

Athlete self-control was assessed through 24 items designed to measure athletes’ emotional regulation during competition (Li and Zhang, 2011). Examples of items are, “In order to complete the training, I can endure extreme fatigue,” “During competition, I always think about the outcome,” and “I can keep calm, regardless of the results.” Items are rated on a 5-point scale, with 1 indicating *completely not describe me* and 5 indicating *completely describe me*. High scores indicate a high level of self-control over emotions. Reliability, as assessed through Cronbach’s alpha, was 0.85 in the current sample.

#### Impulse Control

Impulse control was assessed using a revised impulse scale (Tan and Guo, 2008) based on the self-control scale developed by Tangney et al. (2004). The former comprises 19 items designed to assess one’s ability to control their impulses, alter their emotions and thoughts, and interrupt undesired behavioral tendencies and refrain from acting on them. The scale has four subscales, namely, impulse control (e.g., “I am good at resisting temptation”), work/study ethic (e.g., “I refuse things that are bad for me”), health habits (e.g., “I have a hard time breaking bad habits”), and deliberate action (e.g., “I change my mind fairly often”). Participants rated each item on a 5-point scale (1 = *not at all* to 5 = *very much*), with a high score indicating a high level of impulse control. Reliability, as assessed through Cronbach’s alpha, was 0.76 for impulse control, 0.81 for work/study ethic, 0.77 for health habits, and 0.80 for deliberate action in the current sample.

#### Athlete Self-Efficacy

Self-efficacy was assessed using the Sport Self-efficacy Questionnaire (Wei et al., 2008). It comprises 15 items that are designed to assess athletes’ self-efficacy regarding training and competition on a 5-point rating scale ranging from 1, indicating *rarely*, to 5, indicating *always*. Example items are, “I can cope and deal with any difficulties during the competition” and “I have confidence to beat stronger opponents during training.” Higher scores indicate a high level of self-efficacy. Reliability, as assessed through Cronbach’s alpha, was 0.96 in the current sample.

#### Athlete Performance Success

We used athlete performance success as a correlate of athletes’ profiles of personality and emotional traits. To assess athlete performance success, participants were asked to report on their performance rankings, and we used this information to approximate the performance success or elite standing among professional athletes. For example, if an athlete had won a medal (gold, silver, or bronze) at an international competition, we considered him or her to have more performance success compared to those who had won a medal at a national competition. We considered participants’ standing or performance success on a continuum scale, with 1 indicating *most elite* or *more performance success* and 5 indicating *non-elite standing* or *less performance success*.

### Analysis Design

Given the exploratory nature of the study, the primary analysis method used was multidimensional scaling (MDS) profile analysis (Davison et al., 1996; Ding, 2006). Since this method of profile analysis is relatively unfamiliar to researchers, we describe it in detail below. More detailed discussions can be found in the references provided.

Traditionally, MDS models have been mainly employed for data reduction, wherein the researcher sought to reduce complex interrelationships between stimuli to a simpler and more understandable form. This use is quite similar to that of factor analysis, and the two techniques can be used to study similar issues. However, in MDS profile analysis, we are interested in identifying latent profiles reflecting patterns of co-occurrence in the personality and emotional traits under inquiry, rather than homogeneous constructs. The MDS profile model represents a subgroup of athletes on a continuous basis, with athletes showing different degrees of “matching” prototypical personality and emotional trait patterns, rather than aligning with a simple “yes or no” category. Moreover, MDS profile analysis is based on the framework of prototype theory (Rosch et al., 1976) in which we attempt to identify prototypical profiles of people while allowing each individual to fit these prototypical profiles to different degrees.

Specifically, in the MDS profile model, dimensions are represented as prototypical profiles (i.e., prototypicality of personality and emotional traits) and the fundamental estimates are scale values for these variables. One of the major goals of MDS profile analysis is to determine the number of prototypical profiles expressed in scale values, which indicate a particular arrangement of variables in the profiles. The idiographic aspect of the model can be assessed using individual profile match indices (PMIs). Each individual’s PMIs are equal in number to the number of profiles. These PMIs reflect the degree to which a person’s observed data tends to match the identified prototypical profile—the closer to the core, the more prototypical. Thus, a positive profile match index indicates that the individual’s observed data tend to manifest the personality and emotional trait patterns represented at the positive end of the profile, while a negative profile match index indicates that the individual’s data tend to exhibit a pattern opposite to the profile. These PMIs are useful for individuals because (1) by correlating these indices with other variables, one can study, for example, whether profiles can predict performance success and (2) one can examine a within-person pattern in that a participant may be more likely to manifest one profile than another; this means that one could evaluate how individuals perceive themselves with respect to these profiles.

A word of caution is warranted: in MDS profile analysis, the actual appearance of a particular profile depends on how variables are listed. Since the positioning of each variable is arbitrary, the physical appearance of the profile can be arbitrarily changed without affecting the level, dispersion, or shape of the profile. As a result, some researchers call the positive end of a profile a “profile” and the low points in the profile a “mirror image of the profile” (Davison, 1994). In addition, individuals can be classified into one of several different prototypical profiles based on the posterior probability.

Technically, the statistical assumptions of the MDS profile analysis are minimal. The estimation of profiles does not require multivariate normality and linearity. The model fit can be assessed using a *Stress* value (ranging from 0 to +∞) (Kruskal, 1964), with 0 indicating a perfect fit. Here, it is important to note that, in addition to MDS profile analysis, regression analyses were performed to examine the association between identified profiles and performance success. Meanwhile, the MDS profile analysis was performed using the MDS procedure in Statistical Analysis System (SAS, 2013), a commonly used statistical package. SPSS (IBM Corp, 2017) was also used for generating plots. Eleven percent of performance success values were missing from the data. Since no information could be used to estimate this missing information, we used listwise deletion to handle the missing values. In addition, since these variables were measured on different scales, all personality and emotional trait variables were standardized to ensure a mean of 0 and a standard deviation of 1 in MDS profile analysis.

### Procedure

During the national Taekwondo competition period, the first author asked coaches to administer batteries of questionnaires comprising the above-mentioned measures to Taekwondo athletes during a break. Participation in the study was voluntary and athletes could withdraw from the study at any time. The study was approved by the university committee on human research (Protocol ID 2019157).

## Results

### Prototypical Profiles

When studying profiles of personality and emotional traits, an explicit theory for predicting trait patterns among professional athletes is often not available or only prematurely specified. Under such circumstances, it can be beneficial to derive profiles of personality and emotional traits without specifying how these trait patterns may differ from one another. In the current study, we compared models of one-profile to four-profile solutions and compared the *Stress* values. The *Stress* values were 0.13, 0.09, 0.07, and 0.06 for the one-, two-, three-, and four-profile solutions, respectively. The difference in the *Stress* value between the two- and three-profile solutions was 0.02. It seemed that the data could be approximated by two profiles without being overfitted. Therefore, we chose a two-dimensional solution as our best model of the data for interpretation and subsequent analysis.

The results of the scale values from a two-profile solution are shown in Table 1.

**TABLE 1:** Scale values of two profiles.

	Profile 1	Profile 2
Extraversion	**1.00**	**0.80**
Agreeableness	−0.08	0.10
Conscientiousness	0.31	−**0.59**
Neuroticism	−0.35	−**0.84**
Openness	0.45	0.47
Physical attack	−**1.14**	**0.55**
Verbal attack	−**1.23**	**0.74**
Anger	−**1.30**	0.39
Hostility	−**1.54**	0.27
Impulse control	**1.86**	−**0.50**
Work/study ethic	**1.81**	−0.03
Health habits	**1.97**	−0.34
Deliberate action	**1.58**	−0.12
Self-efficacy	**1.45**	0.26
Depression	−**1.54**	−**0.64**
Loneliness	−**1.62**	−**0.82**
Envy	−**1.52**	−0.37
Athlete self-control	−0.12	**0.69**

*All variables are standardized. Bold indicates a marker variable used to define the profiles.*

The scale values were used to define profiles, which reflected patterns of co-occurrence among personality and emotional traits under inquiry, rather than homogenous constructs. Since the dimensional solution was standardized, we regarded any variable with a scale value greater than 0.5 (i.e., half a standard deviation from the mean) as a salient variable that defined a profile. For example, in Table 1, extraversion has a scale value of 1 on Profile 1, and this variable was considered a salient variable defining this profile. Thus, a profile was is characterized by a combination of different salient or marker variables, and a different combination of salient variables formed a different profile.

**FIGURE 1:**
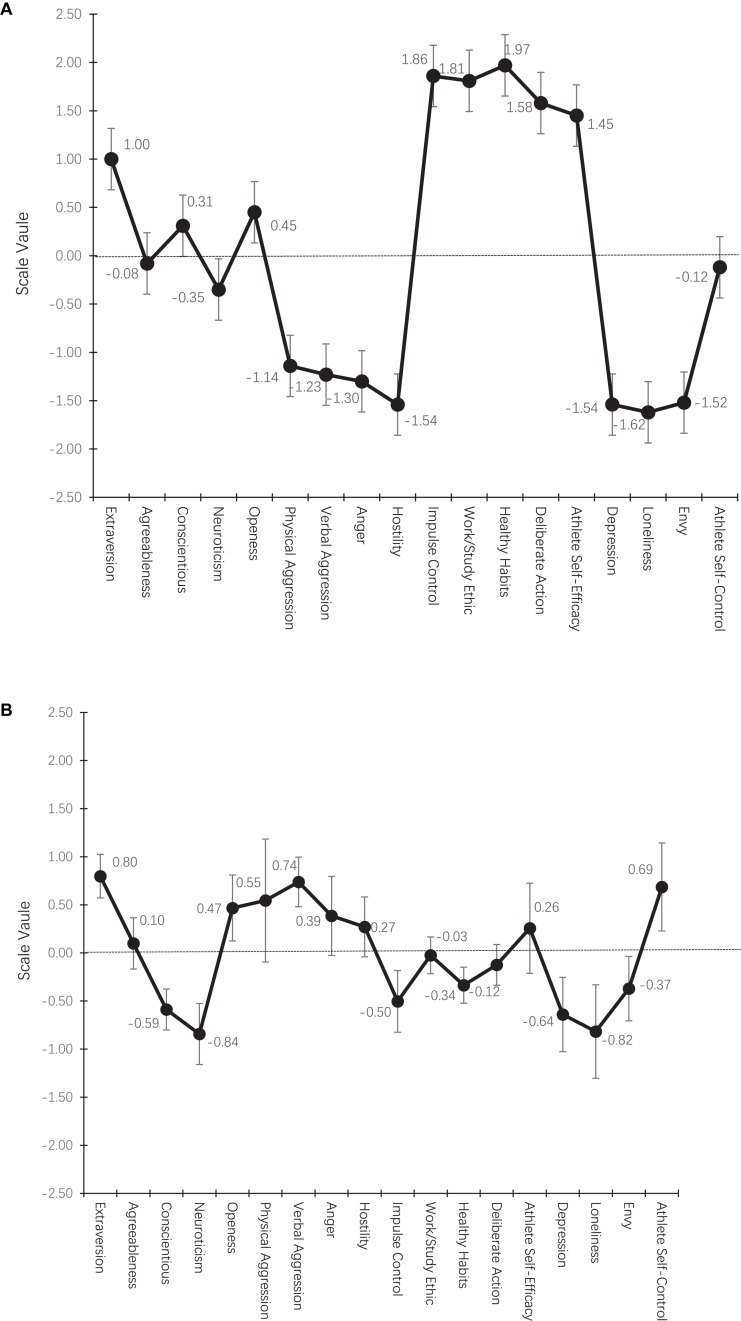
Two profiles of personality and emotional traits. **(A)** Profile 1 is defined by athlete self-efficacy, deliberate action, extraversion, healthy habits, impulse control, and work ethic at the positive end and by anger, depression, envy, hostility, loneliness, verbal attack, and physical attack at the negative end. **(B)** Profile 2 is defined by athlete self-efficacy, extraversion, verbal attack, and physical attack at the positive end and by conscientiousness, depression, loneliness, neuroticism, and impulse control at the negative end. The vertical bar indicates 95% confidence interval.

The scale values in Table 1 are plotted in Figure 1.

As shown in Figure 1A and Table 1, Profile 1 was defined by athlete self-efficacy, deliberate action, extraversion, healthy habits, self-impulse control, and work ethic at the positive end of the profile. Meanwhile, the negative end of the profile was defined by anger, depression, envy, hostility, loneliness, verbal aggression, and physical aggression. Profile 1 revealed that there were two sub-types of athletes: one resembled the profile pattern of Profile 1 at the positive end, and these athletes showed a pattern of elevated athlete self-efficacy, deliberate action, extraversion, healthy habits, self-impulse control, and work ethic; on the other hand, the second sub-type resembled the profile pattern of Profile 1 at the negative end, and the athletes showed a pattern marked by elevated anger, depression, envy, hostility, loneliness, verbal aggression, and physical aggression. The results of our classification analysis based on posterior probability identified 168 individuals as fitting the Profile 1 type, with some athletes more likely to manifest positive self-control behaviors and others more likely to manifest negative emotions and aggressive behaviors.

Profile 2 was defined by athlete self-efficacy, extraversion, verbal aggression, and physical aggression at the positive end, and by conscientiousness, depression, loneliness, neuroticism, and impulse control at the negative end (see Figure 1B). Profile 2 also showed two sub-types of athletes, namely: those who resembled a profile pattern at the positive end, showing elevated levels of athlete self-efficacy, extraversion, verbal aggression, and physical aggression and those who resembled a profile pattern at the negative end, showing elevated levels of conscientiousness, depression, loneliness, neuroticism, and impulse control. In contrast to Profile 1, Profile 2 was less pronounced or more subdued; that is, athletes resembling Profile 2 seemed to have less elevated patterns of personality and emotional traits compared to those resembling Profile 1, who tended to demonstrate more pronounced patterns. In total, 126 athletes were identified as fitting the Profile 2 type. For example, if athletes reported a higher level of self-efficacy, extraversion, verbal aggression, and physical aggression, then the pattern at the positive end of Profile 2 was more prototypical of them.

In sum, the above results show some possible prototypical types of professional Taekwondo athletes with particular personality and emotional traits. As prototype theory suggests (Rosch et al., 1976) there are differences among athletes relative to their centrality of prototypical profile types—some athletes are more prototypical than are others. As an example of prototype logic in this context, if athletes who had won medals (i.e., more successful athletes) were expected to be more likely to manifest the personality and emotional traits represented by Profile 1 than those who had not won any medals, then it can be said that those personality and emotional traits are more typical of athletes who won medals. Such information can be used to investigate the association between profile types and performance success or standing.

### Prototypical Profiles Associated With Performance Success

To investigate the association between profiles and performance success, we conducted a regression analysis with performance success as a criterion variable and derived profiles as predictors. First, we examined whether the results differed across sex; that is, whether the effect of profiles on performance success was moderated by sex. The results showed that there was no significant main effect for sex (*b* = −0.11, *p* = 0.31, partial *h*^2^ = 0.003) nor was there any interaction between Profile 1 by sex (*b* = 0.40, *p* = 0.12, partial *h*^2^ = 0.007) and Profile 2 by sex (*b* = −0.11, *p* = 0.59, partial *h*^2^ = 0.000). Given these results, we dropped the variable of sex from subsequent analyses, using two profiles as the predictors across sex.

The results of subsequent regression analysis showed that the two profiles explained 3% of the variance [*R*^2^ = 0.03, *F*(2,291) = 4.55, *p* = 0.011]. Performance success was not statistically significantly associated with Profile 1 (*b* = −0.12, *p* = 0.371, partial *h*^2^ = 0.00), but Profile 2 significantly predicted performance success (*b* = 0.28, *p* = 0.005, partial *h*^2^ = 0.03). Specifically, the results seemed to suggest that athletes who resembled the positive end of Profile 2 were more likely to have less performance success, while athletes who resembled the negative end of Profile 2 were more likely to have more performance success.

**FIGURE 2:**
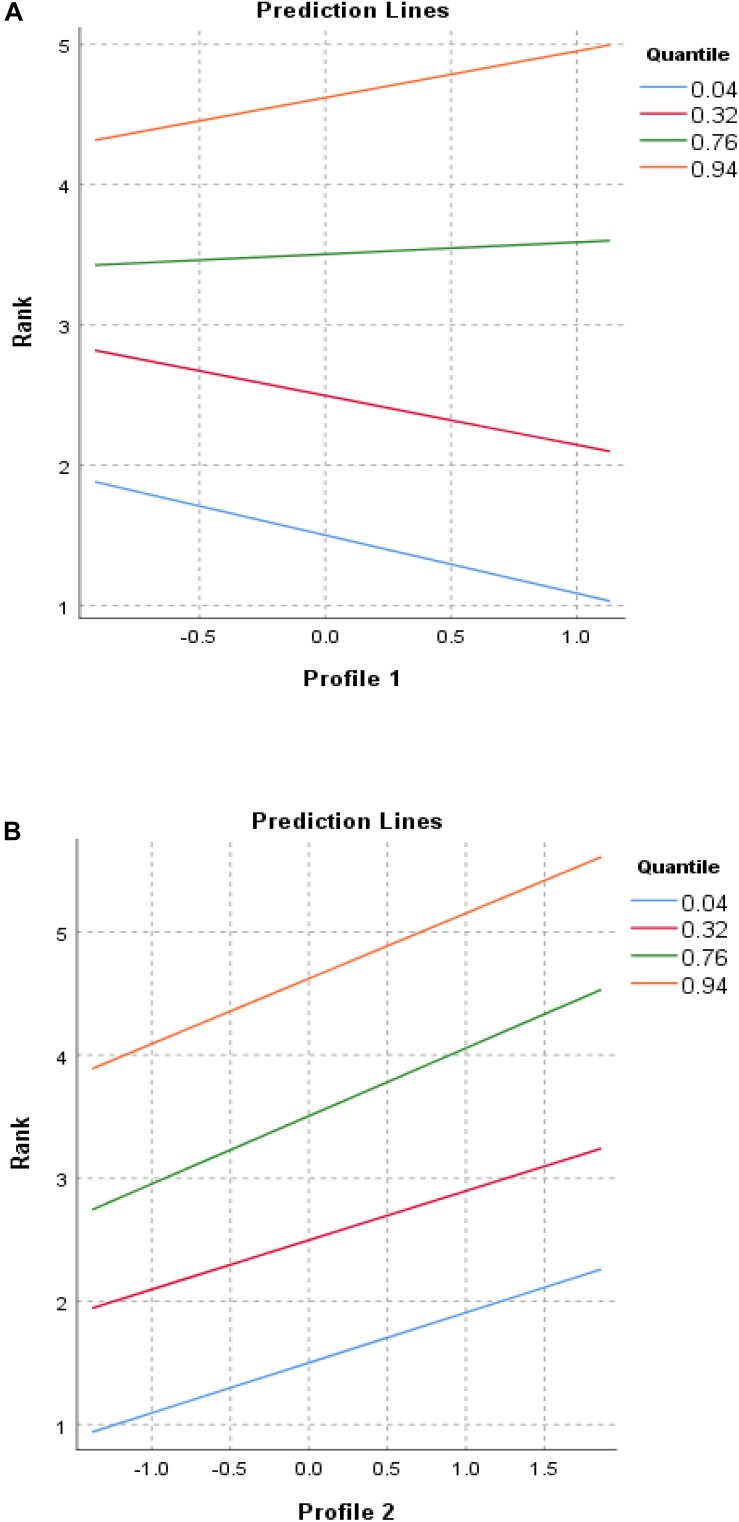
Association between performance success or standing and **(A)** Profile 1 and **(B)** Profile 2. Value on *x*-axis is a profile match index, with a positive number indicating the positive end of the profile and a negative number indicating the negative end of the profile.

In order to get a better picture of what these results indicated, we further performed a quantile regression analysis in which the dependent variable of performance success was modeled at quantiles that corresponded to performance success standing. Specifically, we modeled performance success at quantiles 4 (corresponds to Level 1, the most elite level), 32 (corresponds to Level 2), 76 (corresponds to Level 3), and 94 (corresponds to Level 4). Figure 2 shows the regression line at each quantile for Profile 1 and Profile 2.

As shown in Figure 2A, although not statistically significant, it was interesting to note that Taekwondo athletes with more performance success were likely to be associated with the positive end of Profile 1 (elevated level of athlete self-efficacy, deliberate action, extraversion, healthy habits, impulse control, and work ethic), while athletes with less performance success, and particularly those without any performance success (indicated by rank = 5), were likely to be associated with the negative end of Profile 1 (elevated level of anger, depression, envy, hostility, loneliness, verbal aggression, and physical aggression). This divergent trend in the association may be a reason to situate Profile 1 as not statistically related to performance success.

In contrast with the above, the results of quantile regression analysis showed that well-performing athletes were more likely to be associated with the negative end of Profile 2 (elevated level of conscientiousness, depression, loneliness, neuroticism, and impulse control), while poorly performing athletes were more likely to be associated with the positive end of Profile 2 (elevated level of athlete self-efficacy, extraversion, verbal aggression, and physical aggression) (range of *b* = 0.40–0.55, *p* = 0.00–0.023 at quantiles 4, 32, 76, and 94), as shown in Figure 2B.

The results of a quantile regression analysis were consistent with those of a regular regression analysis, but the findings revealed more detailed information regarding how performance success was associated with different personality and emotional trait profiles.

## Discussion

### Analyzing the Results

The purpose of the current study was (1) to identify personality and emotional trait profiles based on a sample of professional Taekwondo athletes and (2) to investigate the association between the identified profiles and performance success. The study was motivated by the questions of (1) whether an athletic personality exists (Allen et al., 2013) and (2) whether an athletic personality, if it does exist, may be related to the mechanism underlying successful athletic performance. Studying profiles of personality and emotional traits among professional athletes may be the first step toward providing valuable information about what constitutes athletic profiles. Unlike previous studies, which only inquired into one or a few of these variables, this study investigated how a group of variables co-occurred (i.e., coalesced as a profile) and their potential association with performance success.

The main findings from our MDS profile analysis seem to support the presence of salient personality and emotional trait profiles among professional Taekwondo athletes. Two profiles were identified, with Profile 1 more pronounced than Profile 2 in terms of the magnitude of scale values. More specifically, Profile 1 revealed two sub-types of athletes, one with salient positive personality and emotional traits (athlete self-efficacy, deliberate action, extraversion, healthy habits, impulse control, and work ethic) and another with negative ones (anger, depression, envy, hostility, loneliness, verbal aggression, and physical aggression). Interestingly, Profile 1 did not have a statistically significant association with athlete performance success or standing. However, the trend in Figure 2A does seem to suggest that a combination of positive personality and emotional traits is likely to be associated with more successful Taekwondo athletes. Profile 1 also revealed that some athletes without any performance success felt depressed, lonely, and envious, and showed elevated aggressive behaviors. In fact, these findings were consistent with those of previous studies in which extraversion, conscientiousness, neuroticism, anger, depression, and anxiety played a role in athletic performance. Notably, the findings also point to a need for psychological intervention to help athletes cope with negative emotions in ways that enable them to live healthier lives, especially given the markedly negative emotional profile of athletes experiencing anger, depression, envy, hostility, loneliness, verbal aggression, and physical aggression.

In contrast, Profile 2 represents profile patterns with a modest level of mixed positive and negative emotional traits, as indicated by the smaller scale values used to define the profile. Patterns of Profile 2 showed a statistically significant association with Taekwondo athletes’ performance success. In particular, athletes who resembled the pattern at the positive end of Profile 2 (athlete self-efficacy, extraversion, verbal aggression, and physical aggression) were more likely to be associated with less performance success, while those resembling the pattern at the negative end of Profile 2 (conscientiousness, depression, loneliness, neuroticism, and impulse control) were more likely to be associated with more performance success. One reason for the inclination toward more success among athletes resembling the pattern at the negative end may be that they showed modest levels of conscientiousness and impulse control. A modest level of conscientiousness, along with a modest level of impulse control, may effectively enable a balanced mix of planned, focused, flexible, and spontaneous behaviors among athletes (Costa and McCrae, 1992), which may be necessary for automatic and effortless execution during competition. However, it should be noted that, although statistically significant, the effect size of an association between Profile 2 and performance success was small and may not have any practical implications.

### Implications for Sport Practice

The study provides a more comprehensive view of the personality and emotional trait profiles of a sample of professional Taekwondo athletes. This information may help sport psychologists design a better intervention to enhance performance success or avoid wasting limited time and resources. For example, the study found that openness and agreeableness did not form part of the profiles, suggesting that these traits may not be central to sport competition. However, positive personality and emotional traits such as athlete self-efficacy, deliberate action, extraversion, health habits, impulse control, work ethic, and conscientiousness may be necessary for an optimal psychological state for performance success. Thus, practitioners may have to enhance these traits among athletes, since they are challenged with providing interventions that improve core psychological functioning relevant to individual athletes. Evidence from this finding may provide information for designing better intervention strategies.

As another example, the results showed the combination of anger, loneliness, depression, and aggressive behaviors as a pronounced profile among some Taekwondo athletes. This finding is consistent with those suggesting that mental health issues, particularly depression, are a common concern affecting both the general and sporting population (Doherty et al., 2016; Lebrun et al., 2018). Some studies have even indicated that depressive symptoms occur more frequently in elite athletes (Roberts et al., 2016) than in the general population. Although the prevalence of depression is unclear among professional athletes (Beable et al., 2017) our results suggest that, along with other negative emotions and aggressive behaviors, depression is a salient issue among professional athletes. Given the fact that these professional Taekwondo athletes are confronted with unique physical and psychological stressors (Rice et al., 2016), such as overtraining, competitive failure, and career dissatisfaction, our study may prove helpful to this population by providing further evidence of symptoms known to accompany common mental problems. To be sure, in light of our findings, sport psychology practitioners may need to develop or revise coping strategies to optimize athletes’ core psychological functioning for competition. The deeper point here for our purposes, however, is that the current study further advances our understanding of the ways in which athletes’ personality and emotional traits co-occur, which may help sport practitioners or coaches recruit and train athletes and advance their competitive edge.

### Limitations

This study had several limitations that should be addressed in subsequent studies. First, the study used 18 personality and emotional trait measures as inputs for the profile analyses. The profiles will necessarily be influenced by the specific variables included in the study. There is a potentially rich array of personality and emotional traits not captured in the study, and the results may be determined by the variables used as inputs. Thus, the study results may not adequately capture the content domain of possible personality and emotional traits.

Second, two prototypical profiles of personality and emotional traits were identified based on the available measures. However, other possible profiles could be identified. The question of whether a two-profile structure actually exists in the professional Taekwondo athlete population cannot be unequivocally answered based on the current data, and the best solution is to determine this through replication studies.

Furthermore, the study was exploratory in nature, and the prototypical profiles were selected on the basis of statistical grounding, rather than theoretical consideration. Thus, researchers should interpret their findings in light of the possibility that other hypothesized profiles of data might exist. In addition, the measures were self-reported, thereby suggesting biases and inaccuracy in the reports.

Third, in the present study, performance success was operationalized as whether Taekwondo athletes had won medals at international or national competitions. To be sure, there may be better ways of defining performance success. Future studies may thus want to explore other key performance success indicators in examining the relationships between performance success and its antecedents. In addition, the data were collected (i.e., participants completed their questionnaires) without any supervision and without the presence of any researchers. Thus, the participants may not have given the questionnaires their best efforts, which may have impacted the quality of the data.

Despite these limitations, as far as we know, the present study is the first to provide a comprehensive picture of how a set of personality and emotional traits co-occur among professional Taekwondo athletes. These limitations do not necessarily invalidate the potential utility of the current study in that the purpose of identifying personality and emotional trait profiles is to examine the potential psychological patterns typical in certain subgroups and not to eliminate all possible mathematically equivalent or even similar profiles. Nevertheless, it is important to recognize that athletes may develop personality and emotional states in ways that are not described here. It is also important for future studies to investigate the association between prototypical profiles of these traits and other important outcome variables (e.g., automatic and effortless execution).

## Data Availability Statement

The datasets analyzed in this article are not publicly available in order to protect the participants’ confidentiality. Requests to access the datasets should be directed to lb1127@swu.edu.cn.

## Ethics Statement

The studies involving human participants were reviewed and approved by The Research Ethics Committee of College of Sports Sciences. The patients/participants provided their written informed consent to participate in this study.

## Author Contributions

BL and CD conceptualized the framework of the study and wrote the manuscript. BL, FF, HS, LG, and FY help collect the data and provide feedback on the manuscript.

## Conflict of Interest

The authors declare that the research was conducted in the absence of any commercial or financial relationships that could be construed as a potential conflict of interest.
